# Use of radar detectors to track attendance of albatrosses at fishing vessels

**DOI:** 10.1111/cobi.12965

**Published:** 2017-09-11

**Authors:** H. Weimerskirch, D.P. Filippi, J. Collet, S.M. Waugh, S.C. Patrick

**Affiliations:** ^1^ Centre National de la Recherche Scientifique – Centre d'Etudes Biologiques de Chizé 79360 Villiers‐en‐Bois France; ^2^ Sextant Technology 116 Wilton Road Wellington 6012 New Zealand; ^3^ Te Papa Museum 55 Cable Street Wellington 6011 New Zealand; ^4^ School of Environmental Sciences University of Liverpool Liverpool L69 3GP U.K.

**Keywords:** biologging, conservation method, long‐line fisheries, vessel monitoring system, bioregistro, método de conservación, pesca con sedal largo, sistema de monitoreo de navíos

## Abstract

Despite international waters covering over 60% of the world's oceans, understanding of how fisheries in these regions shape ecosystem processes is surprisingly poor. Seabirds forage at fishing vessels, which has potentially deleterious effects for their population, but the extent of overlap and behavior in relation to ships is poorly known. Using novel biologging devices, which detect radar emissions and record the position of boats and seabirds, we measured the true extent of the overlap between seabirds and fishing vessels and generated estimates of the intensity of fishing and distribution of vessels in international waters. During breeding, wandering albatrosses (Diomedea exulans) from the Crozet Islands patrolled an area of over 10 million km^2^ at distances up to 2500 km from the colony. Up to 79.5% of loggers attached to birds detected vessels. The extent of overlap between albatrosses and fisheries has widespread implications for bycatch risk in seabirds and reveals the areas of intense fishing throughout the ocean. We suggest that seabirds equipped with radar detectors are excellent monitors of the presence of vessels in the Southern Ocean and offer a new way to monitor the presence of illegal fisheries and to better understand the impact of fisheries on seabirds.

## Abstract 2

*Article impact statement*: New logger fitted on threatened albatrosses detects and locates radar emissions of boats to estimate fisheries extent in the Southern Ocean.

## Abstract (es)

El Uso de Radares para Rastrear la Concurrencia de Albatros en Navíos Pesqueros

**Resumen**

A pesar de que las aguas internacionales cubren más del 60% de los océanos, el conocimiento sobre cómo las pesquerías en estas regiones le dan forma a los procesos del ecosistema es sorprendentemente pobre. Las aves marinas buscan alimento en los navíos de pesca, lo que presenta efectos potencialmente dañinos para su población. Aun así, la extensión de la coincidencia en el tiempo y el comportamiento en relación con los barcos no se conoce ampliamente. Con el uso de aparatos novedosos de bioregistro, los cuales detectan emisiones de radar y registran la posición de los barcos y las aves marinas, medimos la verdadera extensión de la coincidencia en el tiempo entre las aves marinas y los navíos de pesca, y generamos estimaciones de la intensidad de pesca y distribución de los navíos en aguas internacionales. Durante la época de reproducción, los albatros errantes (Diomedea exulans) de las Islas Crozet patrullaron un área de más de 10 millones de km^2^ a distancias de hasta 2500 km desde la colonia. Hasta el 79.5% de los registradores de datos adjuntos a las aves detectaron navíos. La extensión de la coincidencia en el tiempo entre los albatros y las pesquerías tiene implicaciones amplias para el riesgo de captura accesoria de aves marinas y revela las áreas de pesca intensa en el océano. Sugerimos que las aves marinas equipadas con detectores de radar son monitores excelentes de la presencia de navíos en el océano del sur y ofrecen una nueva manera de monitorear la presencia de pesca ilegal y de mejorar el entendimiento del impacto de la pesca sobre las aves marinas.

## Introduction

There is a serious concern about the potential impact of fisheries bycatch on marine megafauna (Lewison et al. 2004; Lewison et al. 2014). Seabirds have been attracted to vessels for centuries (Coleridge 1895), before the development of industrial fisheries. Today, they attend fishing vessels in large numbers to feed on offal or bait, where their high mortality is the main threat to populations worldwide (Croxall et al. 2012; Phillips et al. 2016). Results of ship‐based studies show how albatrosses react to the presence of vessels (Hudson & Furness 1989; Weimerskirch et al. 2000), and the use of Argos transmitters or Global Positioning System (GPS), combined with vessel‐monitoring‐system (VMS) data from fishing vessels (Witt & Godley 2007; Votier et al. 2010), allowed quantification at an individual level of attendance pattern to vessels and behavioral responses (Granadeiro et al. 2011; Torres et al. 2011; Bodey et al. 2014; Collet et al. 2015).

Interactions with vessels can only be detected from declared vessels whose position is occasionally known within exclusive economic zones (EEZs), whereas in the high seas the position of boats is generally not known (Witt & Godley 2007). Thus, little information is available on the fine‐scale attendance of seabirds outside EEZs (i.e., 66% of the oceans), and limited information is available within EEZs. Being able to detect the presence of vessels throughout a species’ range is essential to derive comprehensive encounter, attendance, and mortality rates (Tuck et al. 2015) and to detect changes in foraging behavior triggered by the presence of vessels. Any change in movement, such as the use of area‐restricted search (ARS) by foraging seabirds, is generally interpreted as an answer to the direct, or indirect, presence of prey (Weimerskirch et al. 2007), but recent evidence shows that change in foraging movements may also occur in the presence of vessels (Torres et al. 2011; Bodey et al. 2014). This has very important implications in terms of interpreting behavior and for conservation because seabird foraging areas are used to propose or designate marine protected areas (Lascelles et al. 2016).

We used newly developed GPS loggers that record radar emissions from vessels. The loggers were fitted on wandering albatrosses (*Diomedea exulans*) foraging from the Crozet Islands. Our aims were to estimate the efficiency of this new technique to detect vessels at sea by comparing radar detections with VMS data of a declared long‐line fishery operating around Crozet and to estimate the extent of overlap with vessels over their entire foraging range of the species.

## Methods

The study was carried out at Possession Island (46°S 51°E), Crozet Islands, in January–March 2015 and 2016. We fitted 53 incubating individuals with XGPS radar loggers: 6 in 2015 and 47 in 2016. Loggers were taped on back feathers. The loggers (35 g, i.e., 0.3–0.4% of the bird's body mass) were well below the recommended mass to avoid potential deleterious effects on the foraging behavior of flying seabirds (Phillips et al. 2003). Birds were caught by hand as they were relieved from their incubation shift by partners and departed to forage. Devices were recovered on their return to the nest after a foraging trip at sea. Forty‐three loggers were recovered from which data were downloaded. The other 10 loggers were either lost at sea (4, detached from back feathers) or were recovered but data could not be obtained (6).

The XGPS logger (Sextant Technology, Wellington, New Zealand) (Supporting Information) was designed to detect interactions between animals and ships at sea by measuring radio emissions in the 9.41GHz X radar band that is used in marine radars. The radar signals emitted from vessels are detected by an omnidirectional microstrip antenna integrating the signal over a programed interval (1 or 2 min or every 5 min). The XGPS is composed of a 77 × 23 × 4 mm main board and an independent 3.7V LiPo battery that is scalable to the species (2000 mAh in this case). The board combines a radar detector, a low‐power Sirf IV GPS, and low‐power NOR FLASH and FRAM memory chips to store the data. The radar signals emitted from the vessel radar are picked up by the loggers with an omnidirectional microstrip antenna tuned at 9.41 GHz  (Supporting Information) connected to a high‐frequency temperature‐compensated Schottky diode acting as a peak detector. The 9.41 GHz radar bursts are converted into a lower frequency signal (3.3V max) proportional to the strength of the radar electromagnetic field the animal is exposed to. The power‐indicator signal could be measured accurately with a fast analogue to digital converter; however, this solution results in excessive power consumption, so instead the power indicator signal is compared sequentially every 100 m with 4 reference voltages (1.65, 0.825, 0.412, and 0.206 V). Every time the power indicator signal is greater than the reference voltage, a digital pulse is generated by a high‐frequency comparator and then counted by the MSP430 microcontroller chip in low‐power mode.

The radar‐level power index is calculated accordingly to the following formula: √(C3^*^8+C2^*^4+C1^*^2+C0),  where C3 is the number of pulses counted by the microcontroller >1.65 V, C2>0.825 V, C1>0.412 V, and C0>0.206 V. The XGPS units were programed to provide locations at 1‐ to 2‐min intervals, giving a 25‐d life span to the battery.

The behavior of birds associated with radar detection was characterized according to movement of birds and radar‐detection patterns. Few successive radar detections (1–5) and no significant change in the route of the bird were categorized as fly pasts. Successive radar detection associated with the linear movement of a flying bird was categorized as follows. Successive radar detections typical of area‐restricted search movements, where the bird alternates between periods of flying and sitting on the water, were categorized as vessel attendance.

We used data from VMS (vessel GPS locations recorded hourly) of French long‐liners operating within the Crozet and Kerguelen EEZ (provided by the Pecheker database hosted at the Museum National d'Histoire Naturelle, Paris [Martin & Pruvost 2007]). The data correspond to 7 vessels fishing under license over the Crozet and Kerguelen shelves and surrounding seamounts. These data and albatross radar‐detection GPS data were imported into Google Earth (https://www.google.fr/earth), which we used to analyze spatiotemporal coincidence of radar detections by XGPS and VMS. Distances between locations of VMS‐equipped boats and bird GPS locations were calculated and associated with intensity of radar signals.

The Préfet des TAAF and Comité de l'Environnement Polaire and CNPN (National Committee for the Protection of Nature) approved the field procedures for our study under IPEV program number 109 (permit number 2015–103, 4 September 2015).

## Results

**Figure 1:**
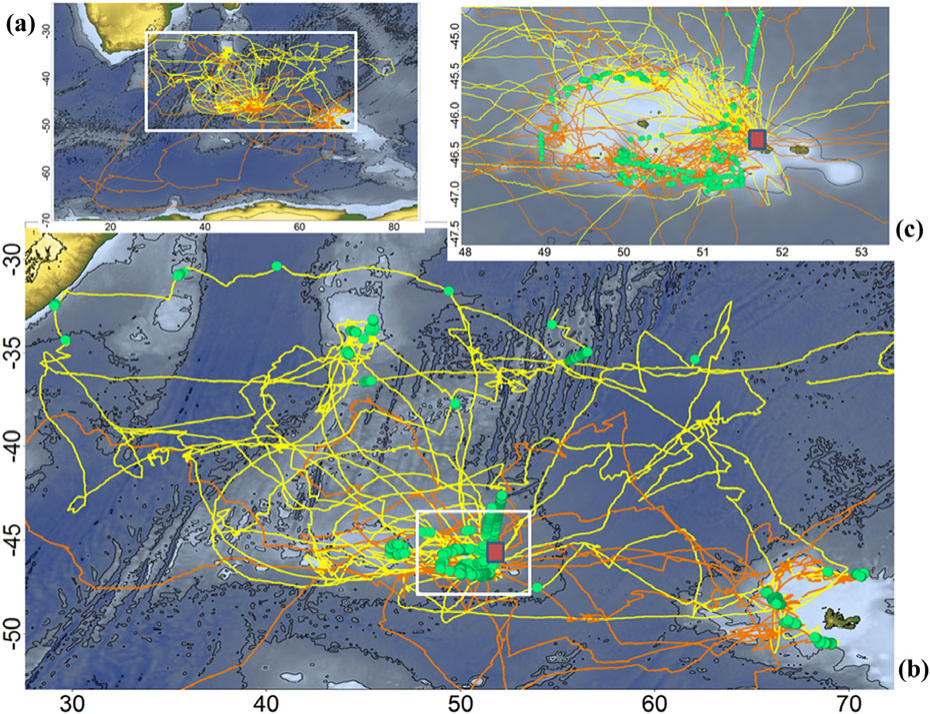
Map of the southern Indian Ocean showing (a) the movement patterns of wandering albatrosses tracked in 2016 (males, orange lines; females, yellow lines), (b) enlargement of rectangular area in (a) showing the movements and location of radar‐equipped vessels (green dots), and (c) enlargement of the Crozet shelf (red square, location of the colony).

Forty‐three foraging trips were recorded with the XGPS in 2015 and 2016, 7 of which were incomplete. The birds traveled between Antarctica and subtropical waters and between the South Africa and central Indian Ocean, covering an estimated 10 million km^2^ (Fig. 1). Over periods of 1 min–23.9 h, 79.5% of the loggers recorded contact with vessel radar (Table 1). Detections were particularly numerous over the Crozet shelf edge (39.6% of detections) but also over the Del Cano rise west of Crozet and the eastern and northern Kerguelen shelf edge (Fig. 1). In these areas, long‐liners fishing for Patagonian toothfish (*Dissostichus eleginoides*) were operating, mostly French vessels for which matching VMS locations were available.

**Table 1:** Types of behavioral movements derived from XGPS radar tracks and radar detection of marine vessels

Behavior	Mean duration (h)	Range (h)	Frequency (%)	Time in contact with radar (%)
Fly past ship	0.03	0.01–0.025	23.9	0.2
Follow cruising ship	2.9	0.20–15.50	8.8	11.4
Attend ship	4.3	0.06–24.90	64.7	45.2

When combining VMS and XGPS data, it appeared that all VMS‐equipped vessels in proximity to birds (<5 km) were detected by the XGPS, except for 1 vessel encountered for a few minutes at >4 km. The distribution of distances between a VMS‐equipped vessel and an XGPS‐equipped bird indicates that radar was detected mainly at a distance of 0.2–2 km and up to 5.5 km (Supporting Information); weaker signals were received at distances >2 km (Supporting Information). The detections other than from VMS‐equipped boats (29%) were recorded to the north of the Crozet Islands over a wide longitudinal band from 38°S to 30°S (Fig. 1), especially over the western Indian Ocean ridge and seamounts south of Madagascar.

The duration of radar contacts (all behaviors combined) represented from 0% to 57.6% of the entire foraging trip (average 6.6 [SD 11.3], *n* = 39). There were no differences between sexes in the proportion of time attending or not attending vessels (*χ* ^2^_1_ = 0.76, *p* = 0.321, Yates corrected, 16 out of 22 females, 16 out of 18 males) or in the type of behavior when attending (*χ* ^2^_3_ Pearson = 4.61, *p* = 0.202). Females interacted with vessels at more northerly latitudes (*F*_1,28_ = 5.4, *p* = 0.025), and at slightly greater distances from the colony (*F*_1,28_ = 3.4, *p* = 0.055) than males (Fig. 1), whereas there were no sex‐specific differences in maximum range or southernmost latitude of an entire foraging trip.

**Figure 2:**
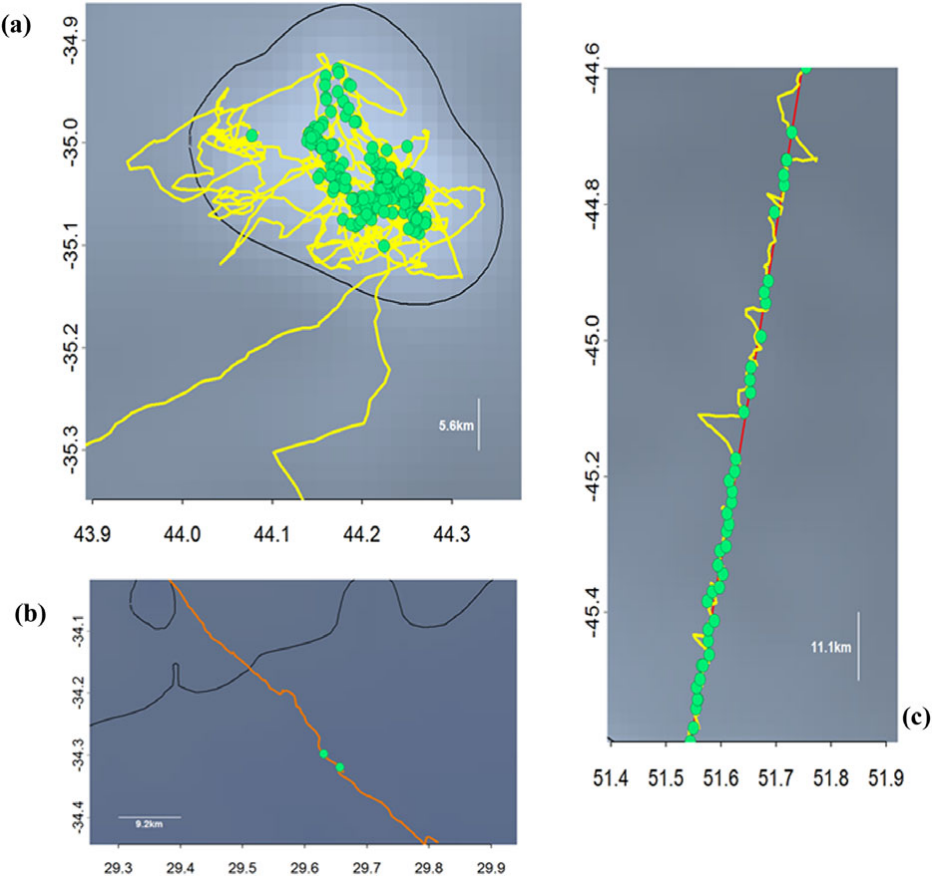
Movement pattern of wandering albatrosses equipped with biologging devices that detect radar emissions and record the position of boats (green dots) and seabirds (yellow and orange lines): (a) attending behavior behind a Japanese fishing vessel (identity determined from Globalfishingwatch.org), (b) fly‐past behavior, and (c) follow behavior (red lines, track of vessel).

The behavior of birds in the presence of vessels can be determined from the GPS track of birds and radar detections (Table 1). Birds either arrived at a vessel and continued on their way (fly past), followed steaming ships (follow), or remained at vessels (attendance) by either continuously sitting on the water nearby or alternating periods of sitting on the water with short bouts in flight, probably to follow a vessel moving between fishing locations (as verified when VMS data were available) (Fig. 2). Fly past represented 23.9% of radar‐detection events. Birds frequently followed steaming vessels; the maximum was 15.5 h of continuous follow during daylight over 334 km (Fig. 2). The most frequent radar detections were associated with attendance behind vessels (Table 1).

## Discussion

Our primary result was that wandering albatrosses from Crozet overlap to a very large extent with vessels in the western Indian Ocean; nearly, 80% of birds had contact with vessels detected by XGPS loggers. This is a minimum estimate because some birds may have encountered vessels at distances >5 km which would not have been detected there. Indeed, wandering albatrosses can change their behavior and approach vessels from distances up to 30 km (Collet et al. 2015). However, once birds have changed their route toward a vessel, they generally approached at close range (<3 km), and XGPS appeared to detect most of these interactions based on the comparison of VMS and XGPS data. Generally, birds spent extended periods behind vessels, suggesting real interactions after attraction, instead of simple spatial overlap (Collet et al. 2015; Collet et al. 2017).

The high encounter rate highlights the propensity of wandering albatrosses to be attracted to vessels. Fishing vessels may operate in traditional foraging zones of albatrosses. The edge of Crozet and Kerguelen Shelves were visited by albatrosses before the development of fisheries and are now also exploited by long‐line fishing vessels (Weimerskirch 1997). The co‐occurrence of vessels and albatrosses over sub‐Antarctic shelf edges does not mean that they are fishing for the same prey because wandering albatrosses mainly feed on squids in these zones (Cherel & Weimerskirch 1999), and the occurrence of Patagonian toothfish in their diet is recent and indicative of opportunistic exploitation of fishery discards (Weimerskirch et al. 1997; Cherel et al. 2017). The reason albatrosses are attracted so strongly to vessels is not clear, particularly because attending sailing vessels has been reported for over 2 centuries. Prior to commercial fishing, there would have been little nutritional reward expected. In the Crozet toothfish fishery, vessels provide feeding opportunities, primarily through discarding of fish waste. The extensive rate of encounter could also be explained by the birds’ opportunistic curiosity or attraction to specific signals such as smell or seabird aggregations (Silverman et al. 2004; Nevitt et al. 2008; Collet et al. 2017).

Over oceanic waters, encounters occurred mainly in subtropical waters, either over seamounts such as those south of Madagascar, or over oceanic waters where there is a high bycatch risk in long‐line tuna fisheries (Tuck et al. 2015). These fisheries represent one‐third of the encounters by Crozet wanderings albatrosses and put females and young age classes of wandering albatrosses that occur there at risk (Weimerskirch et al. 2014). Our results also demonstrate that males and females interacted with vessels in distinct areas. Males interacted mainly with vessels over the edges of the Crozet, Kerguelen and del Cano Shelves, close to the colony, whereas females additionally encountered many vessels over subtropical oceanic waters that are their traditional foraging grounds (Weimerskirch et al. 2014). These sex‐ and age‐specific differences have considerable consequences in terms of conservation because no seabird bycatch mitigation is implemented in subtropical long‐line fleets (Anderson et al. 2011), contrary to those operating in sub‐Antarctic waters. Our findings support observed higher mortality in breeding females, which has far‐reaching demographic consequences (Weimerskirch et al. 2014) and that young birds have high mortality rates during the juvenile and immature phase (Fay et al. 2015).

The XGPS worked efficiently to detect the presence of vessels; all but one of the VMS‐equipped vessels approached within 5 km was detected. Vessels actively fishing can be easily distinguished from cruising vessels because albatrosses attending a vessel during fishing appear to have very sinuous ARS movements over a restricted area with radar detections (Fig. 2), which is different from less‐tortuous large‐scale ARS movements under natural foraging conditions (Weimerskirch et al. 2007). We found that albatrosses encountered fishing vessels over a wide range of the ocean basin, where fleets from many countries operate and whose distribution is generally known only at coarse resolution from regional fisheries organizations (Witt & Godley 2007; Tuck et al. 2015). Thus, the XGPS is a promising tool to not only study the foraging behavior of seabirds in the presence of vessels, but also to detect vessels in particular areas. Given the large direct and indirect impacts fishing vessels have on seabirds (Votier et al. 2004; Pauly et al. 2005; Cury et al. 2011; Bicknell et al. 2013), these devices could become a crucial tool for monitoring marine ecosystems. The ongoing development of XGPS, which can be relayed by Argos or Iridium systems, will allow real‐time monitoring of the presence of vessels anywhere in the range of seabirds, which could thus become patrollers of the southern ocean, allowing better monitoring of fisheries and seabird–fishery interactions. For example, on 1 occasion in the EEZ around Crozet, an XGPS‐equipped albatross detected an undeclared radar signal (i.e., probably an illegally fishing vessel). With such an integrated communication system, it could thus potentially inform authorities in real time of the location of illegal fishing vessels.

Presently, there is an extensive effort to estimate the degree of overlap between seabirds, particularly albatrosses and petrels, and fisheries, especially long‐line fisheries that operate over entire oceanic basins (i.e., tuna fisheries), which represents a primary threat for these seabirds (Croxall et al. 2012). This effort has only been able to estimate potential overlap between fisheries of Regional Fisheries Management Organisms (RFMOs) or national fisheries (Richard & Abraham 2015) and seabirds at very course resolution. In the Indian and Atlantic Ocean, RFMOs provide long‐line fishing effort at the scale of 5° squares of longitude and latitude, which is obviously insufficient to measure overlap and inform conservation measures. With the deployment of XGPS at large scales, it may be possible to measure exactly the overlap with fisheries for each population for which loggers have been deployed. Such data can be used to estimate interactions at the population or individual level (according to sex and age) and therefore improve understanding and measurement of the effects of fisheries on seabird populations. Furthermore, our approach is fishery independent and covers the ecological scale of risk to individual birds. The impact of fishing described for seabirds also applies broadly to other marine megafauna such as marine mammals and turtles (Hays et al. 2016), and our approach may have some utility for these taxa as well.

## Supporting information

An image of the XGPS microstriped antenna (Appendix S1), a finite‐element conceptualization of the XGPS XYZ radiation pattern (Appendix S2), the distribution of radar detections recorded (Appendix S3), and the intensity of the radar signal recorded (Appendix S4) are available online. The authors are solely responsible for the content and functionality of these materials. Queries (other than absence of the material) should be directed to the corresponding author.
