# Supplementary material for: Use of radar detectors to track attendance of albatrosses at fishing vessels
Source: Conserv Biol. 2017 Sep 11;32(1):240–5. doi: 10.1111/cobi.12965 (PMC5811893; doi:10.1111/cobi.12965)
Supplement: Supplementary file 1 — An image of the XGPS microstriped antenna (Appendix S1), a finite‐element conceptualization of the XGPS XYZ radiation pattern (Appendix S2), the distribution of radar detections recorded (Appendix S3), and the intensity of the radar signal recorded (Appendix S4) are available online. The authors are solely responsible for the content and functionality of these materials. Queries (other than absence of the material) should be directed to the corresponding author. [file COBI-32-240-s001.doc]

**Supplementary information**


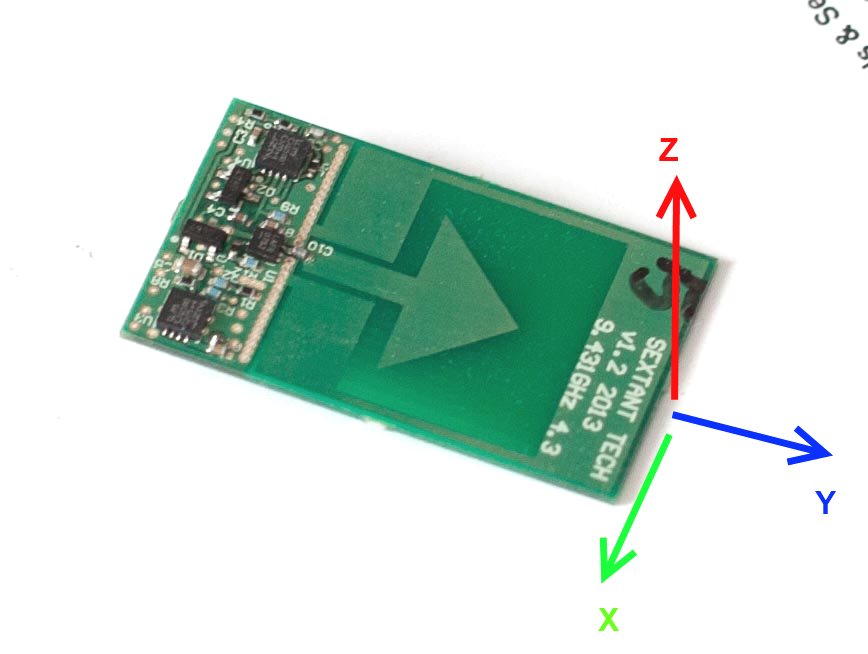

Figure SI 1. XGPS micro-striped antenna


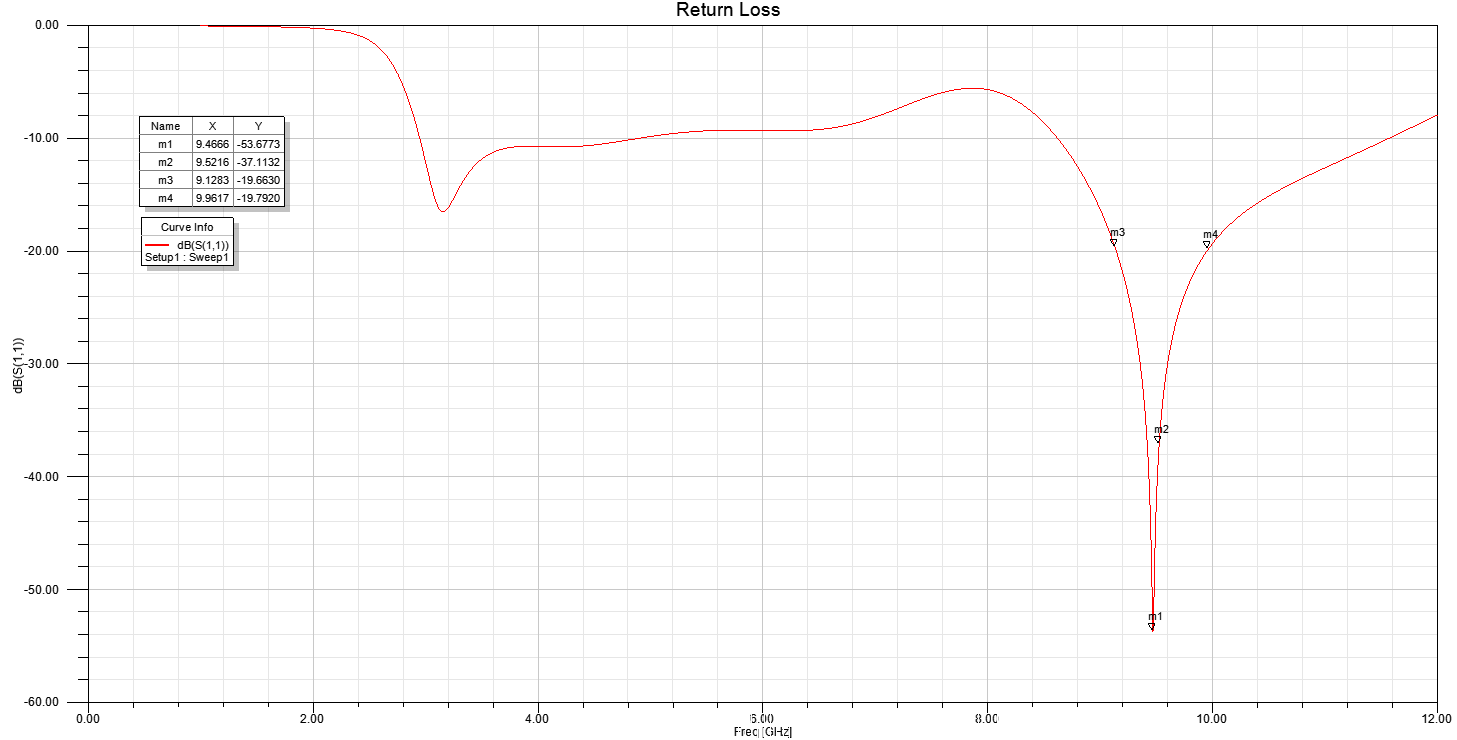


Fig SI 2: Finite element simulation of XGPS antenna return Loss


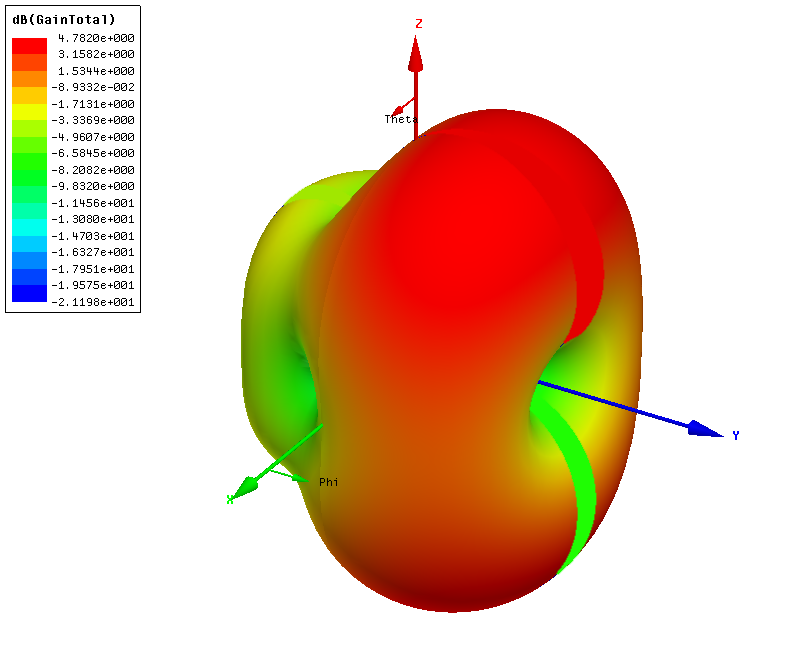


Fig SI 3:  Finite element simulation of the XGPS XYZ radiation pattern


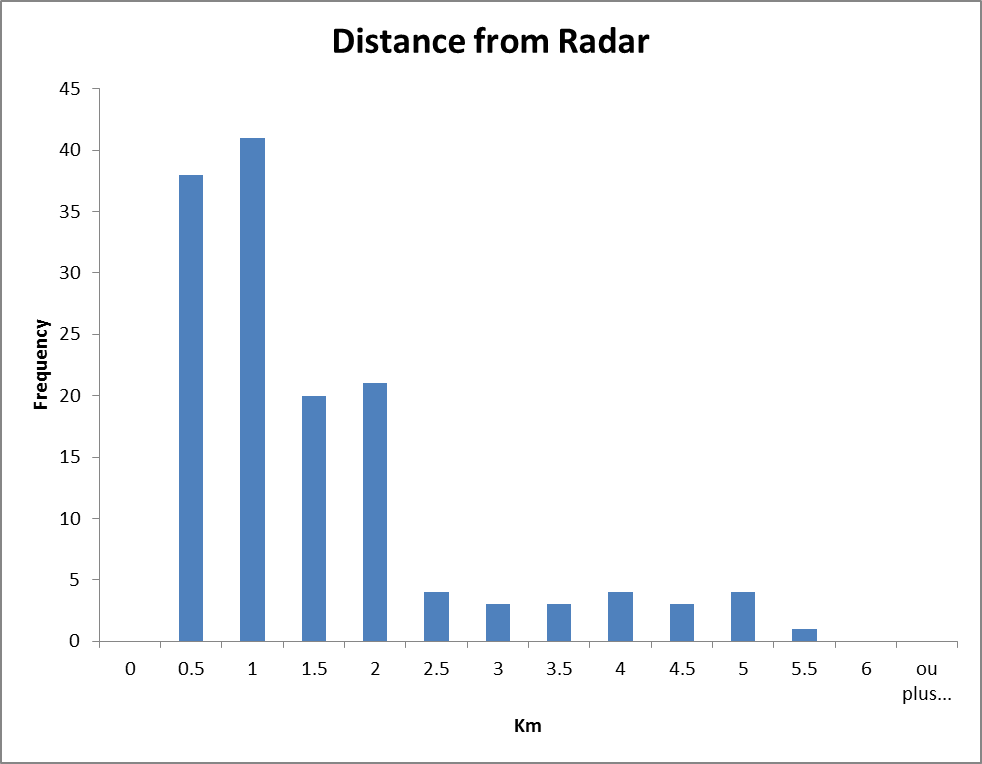


Fig. SI 4 – Distribution of radar detections recorded on the XGPS in relation to the distance between the XGPS and the long-line fishing vessel equipped with VMS.


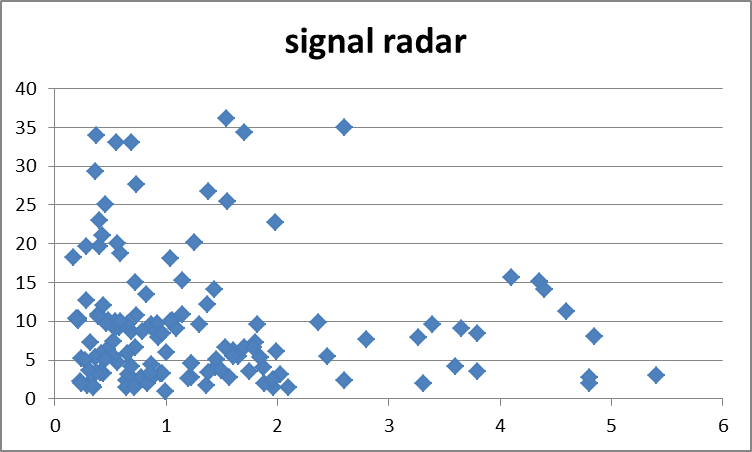


Figure SI 5 – Intensity of the Radar signal recorded by the X GPS in relation to the distance (in km) between the XGPS and fishing vessels equipped with VMS
